# Regional Differences in the Small Intestinal Proteome of Control Mice and of Mice Lacking Lysosomal Acid Lipase

**DOI:** 10.1021/acs.jproteome.4c00082

**Published:** 2024-02-29

**Authors:** Valentina Bianco, Monika Svecla, Giovanni Battista Vingiani, Dagmar Kolb, Birgit Schwarz, Martin Buerger, Giangiacomo Beretta, Giuseppe Danilo Norata, Dagmar Kratky

**Affiliations:** † Gottfried Schatz Research Center, Molecular Biology and Biochemistry, Medical University of Graz, Neue Stiftingtalstrasse 6/4, 8010 Graz, Austria; ‡ Department of Pharmacological and Biomolecular Sciences, Università degli Studi di Milano, Via Balzaretti 9, 20133 Milan, Italy; § Core Facility Ultrastructural Analysis, Medical University of Graz, 8010 Graz, Austria; ∥ BioTechMed-Graz, 8010 Graz, Austria; ⊥ Gottfried Schatz Research Center, Cell Biology, Histology and Embryology, Medical University of Graz, 8010 Graz, Austria; # Department of Environmental Science and Policy, Università degli Studi di Milano, 20133 Milan, Italy; ∇ Centro SISA per lo studio dell’Aterosclerosi, Ospedale Bassini, 20092 Cinisello Balsamo, Italy

**Keywords:** duodenum, jejunum, ileum, proteomics, intestinal metabolism, lysosomal acid lipase, macrophages, inflammation, Trem2

## Abstract

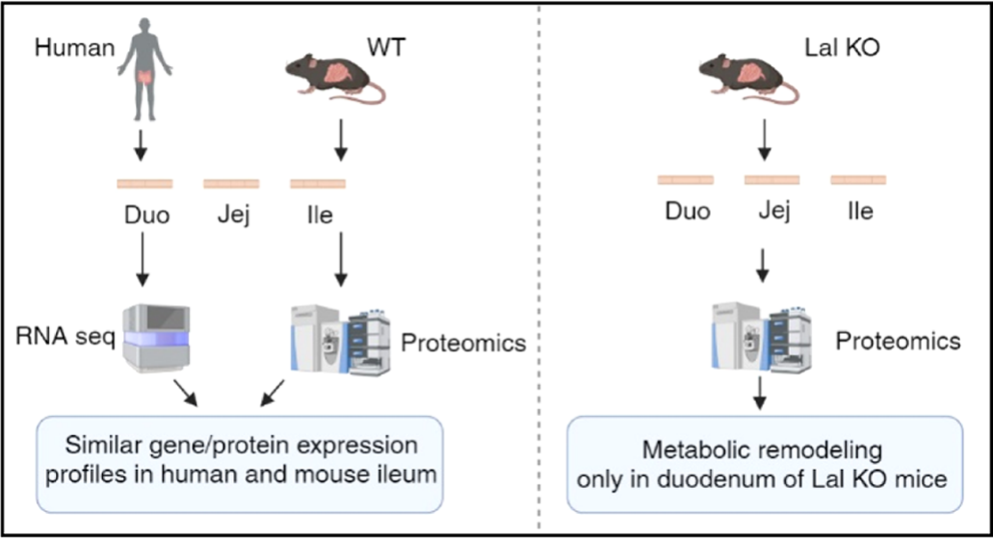

The metabolic contribution of the small intestine (SI) is still unclear despite recent studies investigating the involvement of single cells in regional differences. Using untargeted proteomics, we identified regional characteristics of the three intestinal tracts of C57BL/6J mice and found that proteins abundant in the mouse ileum correlated with the high ileal expression of the corresponding genes in humans. In the SI of C57BL/6J mice, we also detected an increasing abundance of lysosomal acid lipase (LAL), which is responsible for degrading triacylglycerols and cholesteryl esters within the lysosome. LAL deficiency in patients and mice leads to lipid accumulation, gastrointestinal disturbances, and malabsorption. We previously demonstrated that macrophages massively infiltrated the SI of Lal-deficient (KO) mice, especially in the duodenum. Using untargeted proteomics (ProteomeXchange repository, data identifier PXD048378), we revealed a general inflammatory response and a common lipid-associated macrophage phenotype in all three intestinal segments of Lal KO mice, accompanied by a higher expression of GPNMB and concentrations of circulating sTREM2. However, only duodenal macrophages activated a metabolic switch from lipids to other pathways, which were downregulated in the jejunum and ileum of Lal KO mice. Our results provide new insights into the process of absorption in control mice and possible novel markers of LAL-D and/or systemic inflammation in LAL-D.

## Introduction

The small intestine (SI), which accounts for almost 75% of the gastrointestinal tract, is responsible for the survival of the entire organism by absorbing and processing nutrients, eliminating toxic substances, and promoting defense against external pathogens.^1,2^ The SI is divided into three distinct segments from the proximal to distal end, namely, duodenum, jejunum, and ileum. Each of these segments fulfills specific functions.^1^ Analysis of the SI from three healthy donors by single-cell RNA sequencing (scRNA-seq) showed that the expression of most genes involved in the transport of fatty acids, glucose, and cholesterol was increased from the duodenum to the ileum,^3^ underscoring the important role of the ileum in the digestive process. The proteome of cytosolic lipid droplets from mouse enterocytes after a dietary fat challenge advanced the knowledge on dietary fat absorption and lipid trafficking,^4^ but no insights were provided on the metabolism of the three intestinal segments. We previously identified 25 enzymes, including lipases, esterases, and amidases, which were specifically enriched in different parts of the SI.^5^ Although scRNA-seq analysis was performed in the mouse SI, the three regions were not clearly separated and investigated.^6^ To the best of our knowledge, no other studies have so far addressed the functional differences between the intestinal parts.

Triacylglycerols (TGs) stored within cytosolic lipid droplets are degraded by neutral lipolysis in the cytoplasm, initiated by adipose triglyceride lipase (ATGL) and completed by hormone-sensitive lipase (HSL) and monoglyceride lipase (MGL) or by lipophagy in an autolysosome at acidic pH.^7^ Lysosomal acid lipase (LAL) is the sole enzyme known to degrade neutral lipids, such as TGs and cholesteryl esters (CEs), in the lysosome or in an autolysosome to generate fatty acids and free cholesterol^8,9^ essential for anabolic and catabolic pathways. Mutations in the LAL-encoding *LIPA* gene result in rare lysosomal storage disorders (LSDs) with the complete or partial absence of LAL activity. In both conditions, the lysosome is unable to degrade CEs and TGs, which accumulate in the organelle and lead to disturbed homeostasis.^10^ One of the most affected tissues in patients suffering from LAL deficiency (LAL-D) is the SI, in which massive lipid accumulation causes gastrointestinal symptoms, such as vomiting, diarrhea with steatorrhea, and abdominal distension.^11^ We and others have shown that the SI of Lal-deficient (KO) mice is severely affected due to pronounced macrophage infiltration.^12−14^ Interestingly, we observed that macrophages, not enterocytes, are the main cell type that accumulated lipids in these mice, suggesting that LAL does not play a crucial role in lipid metabolism in enterocytes.^14^ The infiltrating macrophages in the duodenum of Lal KO mice displayed a triggering receptor expressed on myeloid cells-2 (Trem2)-like gene signature previously identified in lipid-associated macrophages (LAMs)^15^ and disease-associated macrophages.^16^

The objective of this study was to investigate metabolic differences and similarities among the three intestinal parts in wild-type (WT) mice using an untargeted proteomics approach. Moreover, we aimed to elucidate the potential mechanisms behind the intestinal phenotype of Lal KO mice and to determine whether the three parts of the SI in LAL-D share a common inflammatory and metabolic signature.

## Experimental Procedures

### Mice

Age-and sex-matched WT and Lal KO mice on the C57BL/6J background were housed in a clean and temperature-controlled environment (22 ± 1 °C; relative humidity, 45–65%) with unlimited access to food and water on a regular 12 h/12 h light–dark cycle. Mice were fed a standard chow diet (11.9% caloric intake from fat; Altromin, Lage, Germany). All experiments were performed in accordance with European Directive 2010/63/EU and approved by the Austrian Federal Ministry of Education, Science, and Research (Vienna, Austria; 2020-0.129.904 and 2020-0.688.125).

### Intestinal Lipid Analysis

Duodena, jejuna, and ilea were isolated from 6 h fasted male WT and Lal KO mice (30–33 weeks old), and TG and CE concentrations were measured, as previously described.^17^

### Electron Microscopy

Duodena, jejuna, and ilea were isolated from 4 h fasted male WT and Lal KO mice (21 weeks of age) and processed, as previously described.^14^

### Sample Preparation and Nano-LC-MS/MS Analysis

Duodena, jejuna, and ilea from 6 h fasted male WT and Lal KO mice (*n* = 6/group, 30–33 weeks of age) were pooled in pairs of 20 mg to obtain three biological replicates per intestinal section and genotype. Each replicate (1/2, 3/4, and 5/6) was measured twice, resulting in six values per genotype. The samples were lysed with 8 M urea and 0.1 M Tris-HCl (pH 8.5) in the presence of protease inhibitors (No. 5872S, 1:100; Cell Signaling Technology, Danvers, MA) for 30 min at 4 °C with constant shaking. Samples were then centrifuged at 14,000 *x g* and 4 °C for 30 min. The supernatant was collected, and proteins were quantified using the Lowry protein assay.

A total of 10 μg of proteins was completely dried in a vacuum concentrator at 45 °C for 45 min. Afterward, the dried protein pellet was resuspended in 10 μL of water plus 10 μL of 50 mM ammonium bicarbonate solution (final pH 8.5), followed by protein reduction with 5 mM DTT for 20 min at 55 °C, as previously described.^18,19^ Proteins were then alkylated at room temperature by incubation with 15 mM iodoacetamide for 30 min in the dark. Trypsin (#T7575-1KT, Merck Millipore, Billerica, MA) digestion with a 1:20 enzyme/protein ratio was performed overnight at 37 °C and terminated by acidification with trifluoroacetic acid (final concentration 1%). Proteolytic peptide mixtures were preconcentrated in an Acclaim PepMap 100 (100 μm × 2 cm C18, Thermo Fisher Scientific) and separated on an EASY-Spray column ES802A (25 cm × 75 μm ID) packed with a Thermo Scientific Acclaim PepMap RSLC C18 (3 μm, 100 Å). The separation was achieved using mobile phase A (0.1% formic acid in water) and mobile phase B (0.1% aqueous formic acid/acetonitrile (2:8)), employing the following elution gradient: 4–28% for 100 min and 28–40% for 10 min, followed by 95% for a total runtime of 150 min, at a flow rate of 300 μL/min. For purification, C18 resin pipet tips were used and the proteolytic peptide mixtures were analyzed in duplicate using a Dionex Ultimate 3000 nano-LC system (Sunnyvale, CA) connected to an Orbitrap Tribrid mass spectrometer (Thermo Scientific, Bremen, Germany) equipped with a nanoelectrospray ion source. Full MS scans were collected in positive ion mode with a resolution of 120,000 (375–1500 *m*/*z* range) in data-dependent mode with a cycle time of 3s between master scans, as previously described.^20^ MS/MS spectra were collected in the centroid mode.

### Data Processing and Analysis

Data were processed and analyzed, as previously described.^21^ Briefly, the raw MS data files were transformed to mzML format (MSconvert tool of the ProteoWizard program, version 3.0.1957; Palo Alto, CA).^22^ MzML files were then analyzed using OpenMS (version 2.64, deNBI, Germany) nodes running on the open-source software platform KNIME (version 4.6, Knime AG, Zurich, Switzerland).^23^ MS/MS spectra were searched against a mouse Uniprot FASTA database (uniprot-mus + musculus.fasta, downloaded from www.uniprot.org, Jan 2022, 17.527 entries) and a common contaminant protein database using the DDASSQ pipeline, as previously described.^21^ The OpenMS PeptideIndexer node was used to index peptide sequences with a defined leucine/isoleucine equivalence. The protein inference analysis was then applied to infer proteins using the default parameters provided by the developers.^24^ Estimates of protein abundance were determined using the FeatureFinderMultiplex node to generate spectral features. Thereafter, protein inference analysis-assisted false discovery rate (FDR)-multiple score estimation and filtering (combined FDR score 0.01), ID mapping and combination with peptide IDs, and subsequent alignment, grouping, and normalization (i.e., MapAlignerIdentification, FeatureUnlabeledQT, and ConsensusmapNormalizer nodes) were performed.^25^ Next, the OpenMS ProteinQuantifier node was employed to calculate the label-free quantification (LFQ) of proteins and peptides based on the intensities of the three most abundantly detected peptides. The corresponding output files were read as CSVreader node output tables and exported to Microsoft Office Excel. LFQ abundances were obtained by comparing WT and Lal KO mice for each intestinal segment. LFQ abundances were log_2_-transformed, and proteins with all replicates were included, except for LAL, where two values were missing in the ileum of WT mice. The abundances were normalized across all of the proteins from each sample by scaling each value against the average of the median. For the abundance distribution width, the slope between the average values was calculated. PCA plots for the different proteomes were made using ClustVis.^26^ Volcano plots were generated using VolcaNoseR.^27^ The heatmaps were created online using Morpheus software (https://software.broadinstitute.org/morpheus). Gene Ontology (GO) and Kyoto Encyclopedia of Genes and Genomes (KEGG) enrichment analyses using the Database for Annotation, Visualization, and Integrated Discovery platform (DAVID; NIAID, Bethesda, MD) and ingenuity pathway analysis (IPA; QIAGEN, Redwood City, CA) were performed as downstream analyses.

### Western Blotting Analysis

Duodenal, jejunal, and ileal samples were lysed with RIPA buffer, and 50 μg of protein was separated by SDS-PAGE and transferred to a PVDF membrane to detect GPNMB (ab188222; Abcam, Cambridge, U.K.). β-actin was used as loading control (A5316; 1:10,000; Sigma-Aldrich, St. Louis, MO). Secondary HRP-conjugated antirabbit (#31460; 1:2500; Thermo Fisher Scientific, Waltham, MA) and antimouse (P0260; 1:1000; Dako, Glostrup, Denmark) antibodies were visualized by enhanced chemiluminescence detection on a ChemiDoc MP imaging system (Bio-Rad Laboratories, Hercules, CA).

### RNA Isolation and Quantitative Real-Time PCR

RNA extraction and quantitative real-time PCR were performed, as previously described.^14^ Samples were analyzed in duplicate and normalized to cyclophilin A mRNA expression as the reference gene. Expression profiles and associated statistical parameters were determined by the 2^–ΔΔCT^ method. The following primers were used: cyclophilin A (forward: 5′-GAGCTGTTTGCAGACAAAGTTC-3′; reverse: 5′-CCCTGGCACATGAATCCTGG-3′) and Trem2 (forward: 5′-CTGGAACCGTCACCATCACTC-3′; reverse: 5′-CGAAACTCGATGACTCCTCGG-3′).

### Plasma TREM2 Measurement

Plasma TREM2 concentrations were quantified with a commercial ELISA kit (#ELM-TREM2-1-RB; BioCat, Heidelberg, Germany) following the manufacturer’s instructions. Samples from 12 h fasted male WT and Lal KO mice (40 and 50 weeks of age) were diluted 1:25.

### Human Data Reanalysis

FASTQ files were obtained from the Gene Expression Omnibus database (accession code GSE185224) and aligned to the mouse reference genome (GRCh38) using the “cellranger count” function (10× Cell Ranger software, version 7.1.0). The filtered_feature_bc-matrix folder with the barcodes.tsv.gz, features.tsv.gz, and matrix.mtx.gz files was created for each donor. To obtain the CSV file with the metadata of the cells, the file “GSE185224_clustered_annotated_adata_k10_lr0.92_v1.7.h5ad.gz” was downloaded and elaborated in Scanpy (Python, version 3.11.3). The functions ‘Read_h5ad’ and ‘var.to_csv’ were used to access the file and extract the metadata into a CSV file, respectively. The “Read10X” function in Seurat (version 4.3.0) in the R environment was used to open the filtered_feature_bc matrix folder of each patient, and the “CreateSeuratObject” function was applied to convert each file into a usable file for Seurat. The metadata were imported in Rstudio (version 4.3.1), and the “AddMetadata” function was used to link the various cells to the respective intestinal region. The three final Seurat files were merged, and the number of genes from the duodenum, jejunum, and ileum was used to obtain the complete gene expression profile. To compare this data set with our proteomics results, the data set was reanalyzed as a pseudobulk, taking into account all cells present in the three intestinal tracts. For Spearman correlation coefficients, the “ggplot2” function from RStudio was applied.

### Statistics

GraphPad Prism (version 9.3.1, GraphPad Software, San Diego, CA) was used for graphical representation and statistical analysis. Data are presented as mean ± SD. Comparison between two groups was performed using unpaired Student’s *t*-test, and comparisons of multiple groups were analyzed by two-way ANOVA, followed by Tukey’s post hoc test. For GO, the results were considered significant for an FDR < 0.05. Significance levels were set as follows: *p < 0.05, **p ≤ 0.01, ***p ≤ 0.001, and ****p ≤ 0.0001.

## Results

### Different Proteomic Profiles between the Duodenum, Jejunum, and Ileum of WT Mice

**Figure 1:**
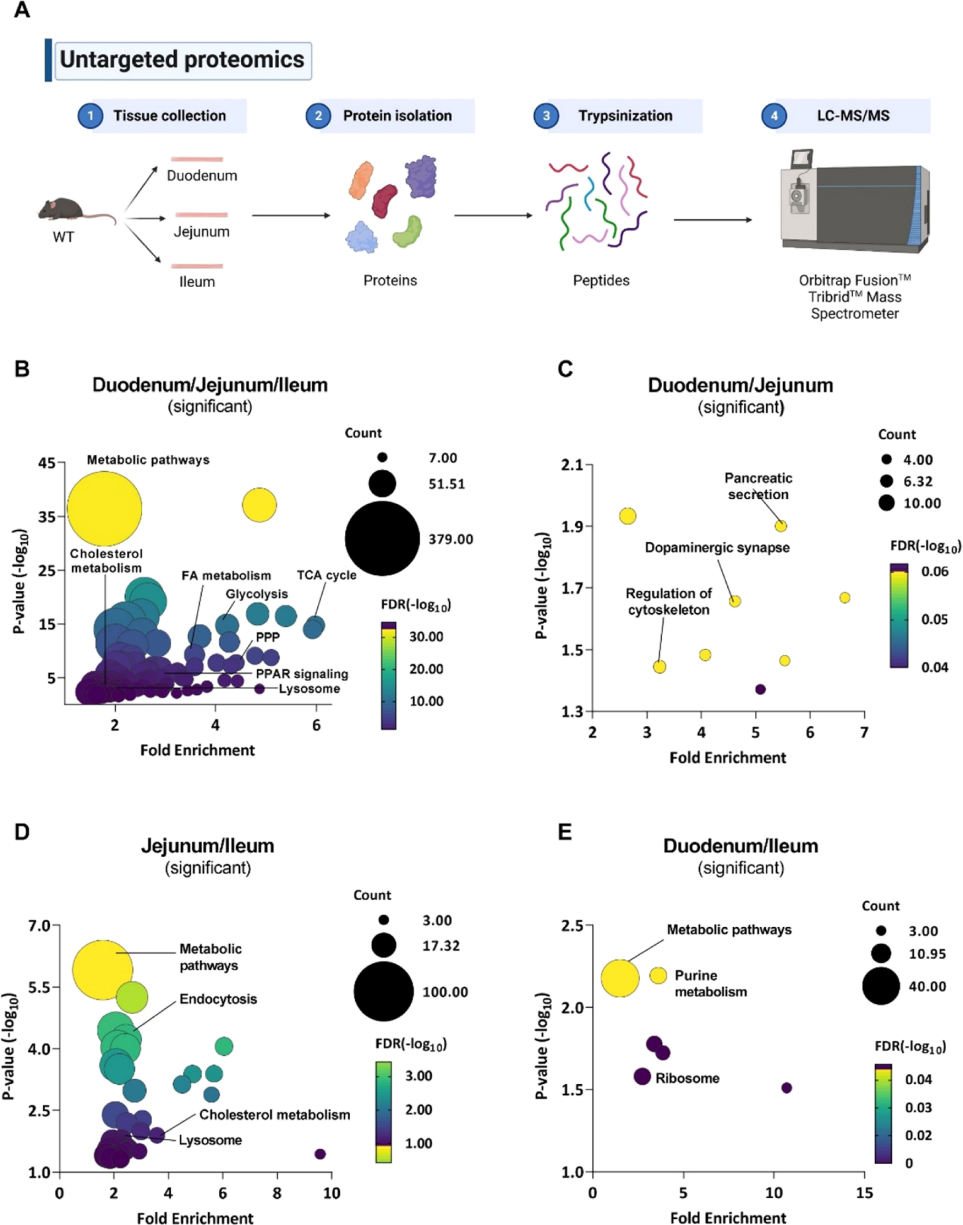
Different proteomic profiles between the duodenum, jejunum, and ileum of WT mice. (A) Untargeted proteomics workflow for sample preparation and LC-MS/MS acquisition. (B) Bubble plot of significantly different proteins between at least two parts of the SI of WT mice. Bubble plots of KEGG analysis of significantly differentially expressed proteins between the (C) duodenum and jejunum, (D) jejunum and ileum, and (E) duodenum and ileum of WT mice.

As a comprehensive knowledge of the differences and similarities between the three intestinal parts is still missing, we started our analysis by comparing the duodenum, jejunum, and ileum of WT mice using untargeted proteomics (Figure 1A). We identified 1930 proteins common to all three parts, 421 proteins shared between the duodenum and jejunum, 756 proteins shared between the jejunum and ileum, and 299 proteins shared between the ileum and duodenum (Figure S1A and Tables S1 and S2). Moreover, 557, 642, and 571 proteins were specific to the duodenum, jejunum, and ileum, respectively (Figure S1A and Table S3). Of the 1930 common proteins among the SI, only 19 were equally expressed in all three intestinal segments (Figure S1B). These proteins are involved in mitochondrial functions, metabolic pathways, and complement and coagulation cascades. With the remaining 1911 significant proteins, we performed KEGG enrichment analysis and observed distinct metabolic profiles among the three SI parts, with differences in oxidative phosphorylation, fatty acid (FA) metabolism, glycolysis, the TCA cycle, and multiple lipid-related pathways (Figure 1B). As an example, we analyzed proteins involved in oxidative phosphorylation (Figure S2A) and associated with the lysosome (Figure S2B) to highlight the different abundance in the three intestinal tracts.

To further investigate the similarities and variations in WT SI, we performed analyses of two intestinal tracts each (Table S2). Between the duodenum and the jejunum of WT mice, there were 129 significantly and 292 nonsignificantly different (and therefore equally expressed) proteins (Figure S3A). Separate KEGG enrichment analysis with significantly (*p* < 0.05) and nonsignificantly (*p* ≥ 0.05) differentially expressed proteins revealed that regulation of cytoskeleton and pancreatic secretion was different (Figure 1C), whereas peroxisome, lysosome, and adherens junctions were comparable between these two tracts (Figure S3B). KEGG enrichment analysis of the 614 significantly different proteins between the jejunum and ileum (Figure S3C) showed that pathways related to endocytosis, cholesterol metabolism, and the lysosome were altered (Figure 1D), whereas the 142 nonsignificantly different proteins were associated with oxidative phosphorylation, ribosome, and regulation of the cytoskeleton (Figure S3D). Comparison of the ileum and duodenum of WT mice revealed 287 proteins to be statistically different (Figure S3E) and linked to purine metabolism and the ribosome by KEGG enrichment analysis (Figure 1E). The remaining 12 (mainly cell surface or mitochondrial) proteins were not significantly different between the WT duodenum and ileum (Figure S3F). Finally, we also analyzed the tract-specific proteins (Table S3) and listed the 20 most abundant proteins for WT duodenum, jejunum, and ileum, respectively (Figure S4A–C).

### Several Metabolic Pathways Share a Comparable Expression Profile in Mouse and Human SI

**Figure 2:**
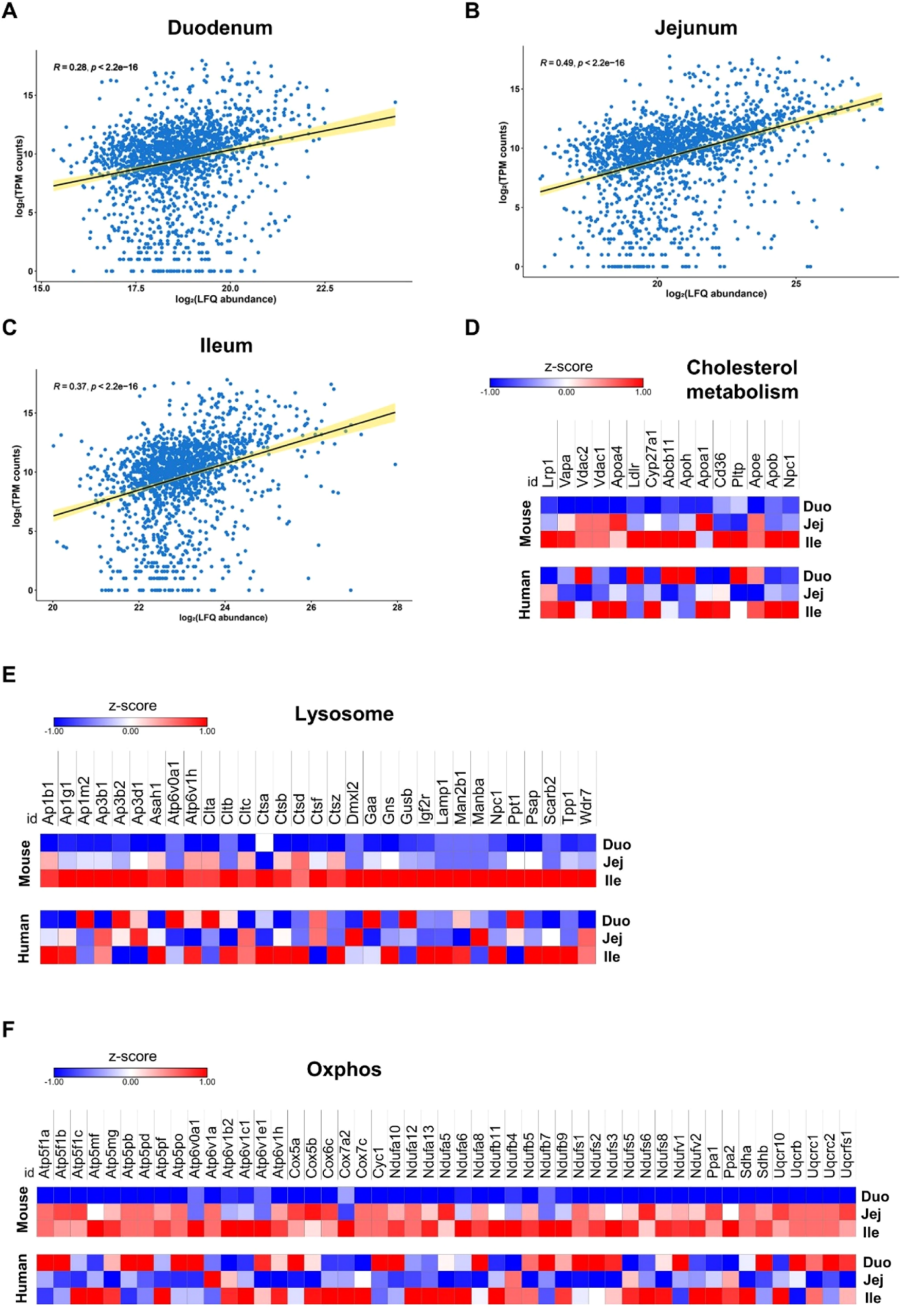
Several metabolic pathways share comparable expression profiles in mouse and human SI. Spearman correlation scatter plots of log_2_ LFQ abundances of the mouse proteome and log_2_ of TPM counts of human single-cell RNA-seq data in (A) the duodenum, (B) the jejunum, and (C) the ileum. Heatmaps of proteins (mouse, top) and genes (human, bottom) involved in pathways related to (D) cholesterol metabolism, (E) lysosome, and (F) oxidative phosphorylation.

To gain a better understanding of the differences between the human and mouse intestine and because proteome data from humans are, to our knowledge, not available, we compared our proteomics data from WT mice with a published scRNA-seq data set of healthy humans.^3^ To allow comparison of the two data sets, we reanalyzed the human scRNA-seq data as a pseudobulk, considering only the separation in duodenum, jejunum, and ileum. We found that the data sets were positively correlated for all intestinal parts (Figure 2A–C). Examination of the expression profiles for several metabolic pathways and organelles, such as the lysosome, oxidative phosphorylation, and cholesterol metabolism, revealed that several proteins highly abundant in the mouse ileum displayed comparable gene expression in the human ileum (Figure 2D–F and Tables S3 and S4). For example, CD36, ApoB, NPC1, and CYP27A1 involved in cholesterol metabolism, cathepsins, and LAMP1 involved in the lysosome, as well as cytochrome oxidases, members of the ATP synthase subunits 5 and 6, and the NADH dehydrogenase families implicated in oxidative phosphorylation followed the same expression profile in mice and humans. Other pathways, including glycolysis, TCA cycle, and FA metabolism, displayed a similar pattern (Figure S5A–C). These results suggest that the abundance of many proteins from the duodenum to the ileum of WT mice is replicated by human intestinal gene expression.

### Distinct Histological and Proteomic Profiles in the Three Parts of the SI of Lal KO Mice

**Figure 3:**
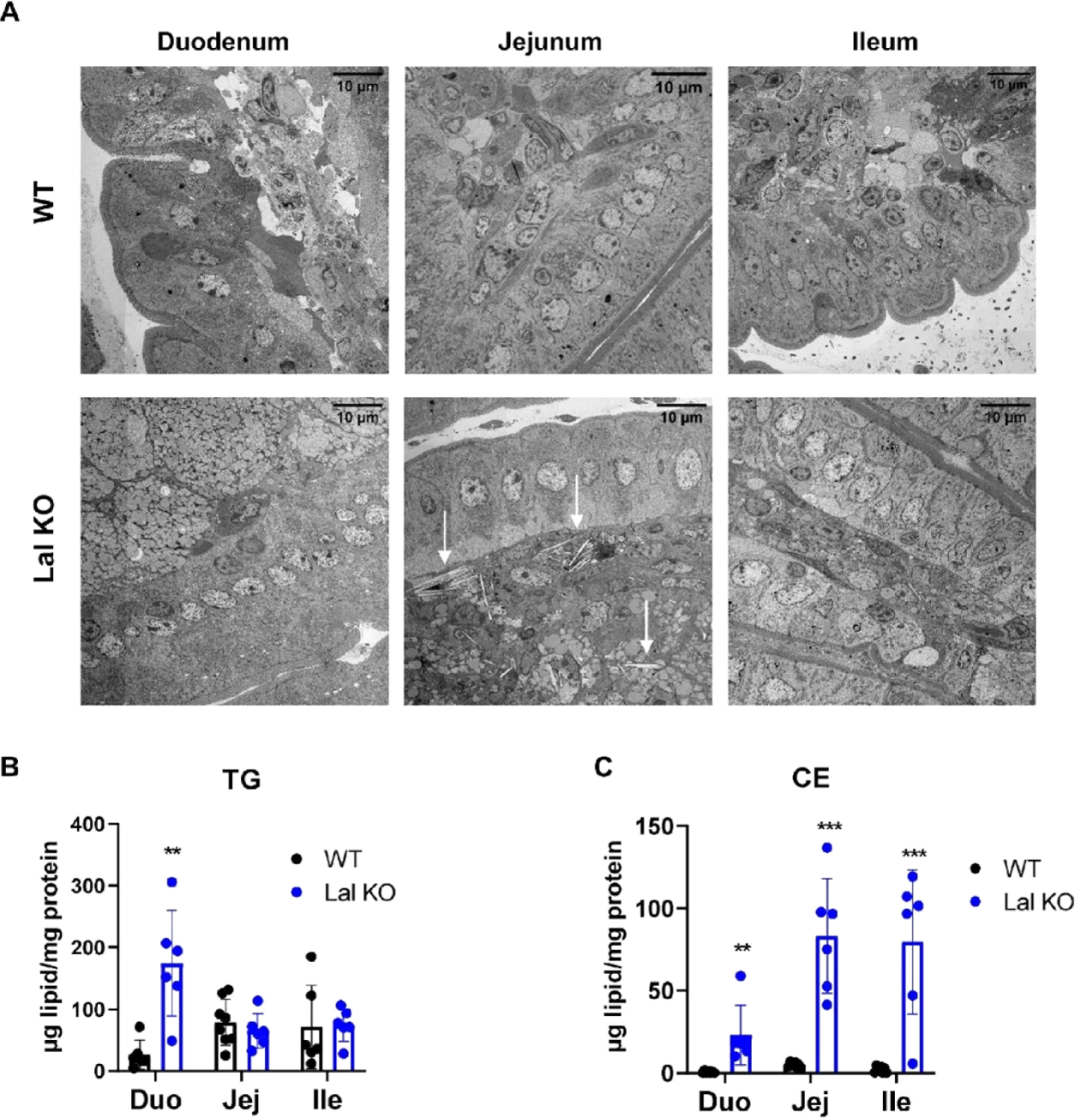
Distinct changes in histological and proteomic profiles in the three parts of the SI between WT and Lal KO mice. (A) Electron micrographs of the duodenum, jejunum, and ileum of 4 h fasted WT and Lal KO mice. White arrows indicate cholesteryl ester (CE) crystals. Scale bar: 10 μm. Quantification of intracellular (B) triacylglycerol (TG) and (C) CE concentrations in 6 h fasted WT and Lal KO mice. Data represent mean ± SD (*n* = 6–8); **p ≤ 0.01; ***p ≤ 0.001.

We have previously shown that the duodenum and jejunum of Lal KO mice are severely affected due to marked macrophage infiltration.^14^ Comparison of proteomics data of the three intestinal parts from WT mice revealed that the ileum had the highest LAL abundance (Figure S6A). To further investigate the intestinal phenotype caused by LAL-D, we first performed electron microscopy of intestinal sections from Lal KO mice. We observed pronounced morphological changes in all three tracts of the SI compared to that in WT mice (Figure 3A). Despite differences between the duodenum, jejunum, and ileum in Lal KO mice, all intestinal sections exhibited tremendous lipid accumulation with varying degrees of macrophage infiltration. The highest number of lipid-laden macrophages was present in the duodenum, followed by the jejunum and ileum. We also identified many CE crystals in the jejunum (Figure 3A), as described previously in the liver of these mice.^13^ The duodenum of Lal KO mice displayed the highest TG accumulation, whereas the jejunum and ileum accumulated more CEs (Figure 3B,C).

To determine the possible cause underlying these differences, we performed untargeted proteomics on the three intestinal parts (Tables S5–S7). Principal component analysis of protein abundance revealed a clear separation of samples between WT and Lal KO mice and an expected variation between individual mice (Figure S6B–D). In agreement with morphological changes, the duodenum had the highest number of differentially expressed proteins (*p* < 0.05), with 428 upregulated (log_2_ (FC) > 0.5) and 346 downregulated proteins (log_2_ (FC) < −0.5) out of 2756 quantified proteins with no missing values in all samples. In the jejunum, we detected 282 upregulated and 363 downregulated proteins out of a total of 3840. In the ileum, 216 of a total of 3186 proteins were upregulated and 178 were downregulated. These results suggest morphological variations between the duodenum, jejunum, and ileum of WT and Lal KO mice, which are reflected in their respective proteomes.

### LAL-D Is Associated with Metabolic Remodeling of the SI

**Figure 4:**
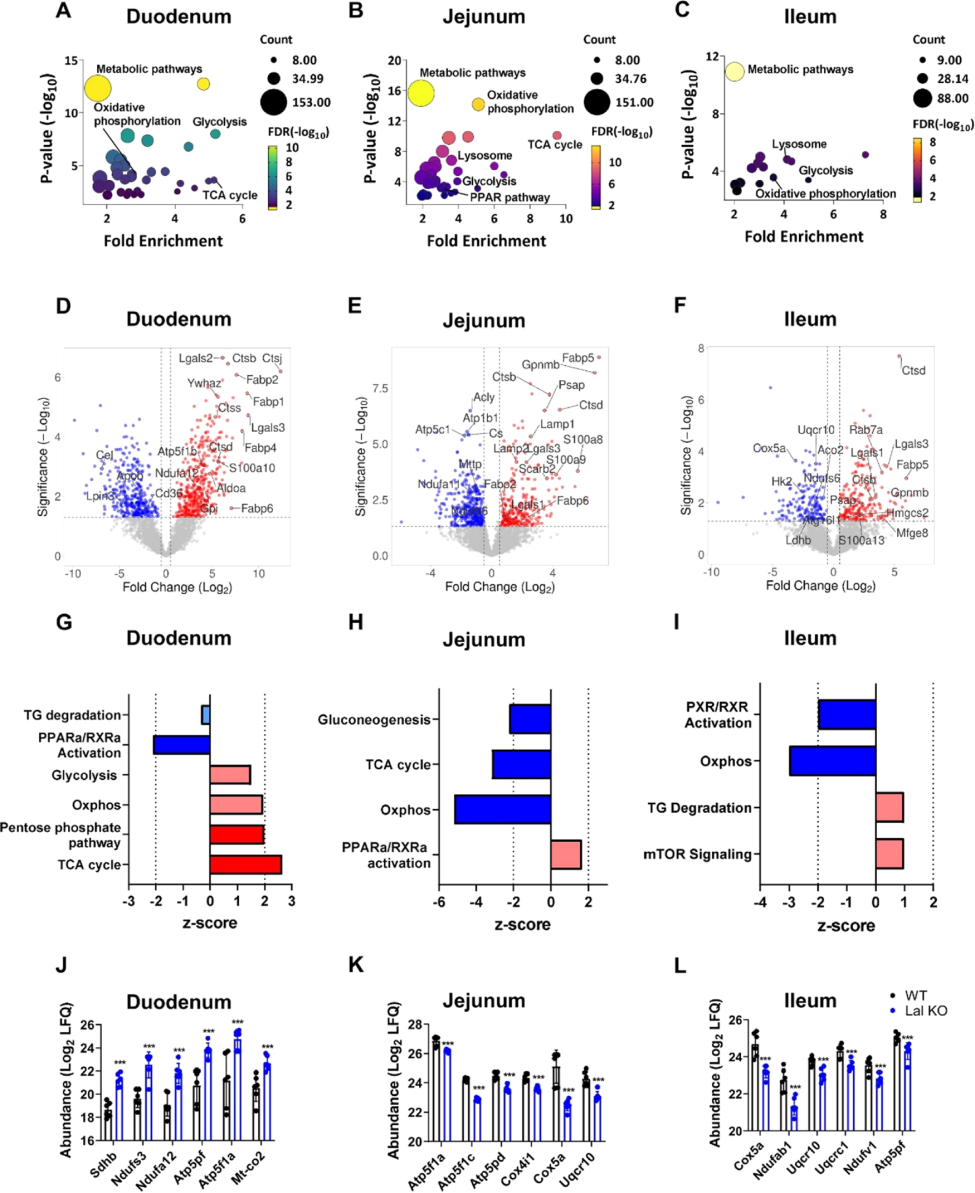
LAL-D is associated with metabolic remodeling of the SI. Bubble plots of KEGG enrichment analysis of the (A) duodenum, (B) jejunum, and (C) ileum of WT and Lal KO mice. Volcano plots of all proteins of the (D) duodenum, (E) jejunum, and (F) ileum of WT and Lal KO mice. Ingenuity pathway analysis (IPA) of enriched canonical pathways related to energy metabolism of differentially expressed proteins in the (G) duodenum, (H) jejunum, and (I) ileum of Lal KO mice. Dark blue represents pathways with a *z*-score < −2; light blue represents pathways with −2 < *z*-score <0; light red represents pathways with 0 < *z*-score <2; dark red represents pathways with *z*-score >2. The LFQ (log_2_-transformed) of the top 6 significant proteins identified by IPA software to be involved in “oxidative phosphorylation” in (J) duodenum, (K) jejunum, and (L) ileum of Lal KO mice. Data represent mean ± SD (*n* = 6); ***p ≤ 0.001.

To investigate the role and molecular function of differentially expressed proteins, we first performed a KEGG pathway analysis, which indicated that metabolic pathways, glycolysis, and oxidative phosphorylation were among the most enriched pathways (FDR < 0.05) in all three parts of the Lal KO SI (Figure 4A–C and Tables S5–S7). Proteins similarly upregulated (*p* < 0.05) in all three intestinal parts of Lal KO mice included lysosomal proteins, such as cathepsins (CTSB, CTSD), inflammatory markers, such as galectins (LGALS) and S100 family proteins (S100a), and fatty acid-binding proteins (FABPS; Figure 4D–F). Moreover, proteins of the various complexes of the electron transport chain, such as ATP synthase subunit β (ATP5F1B) and NADH dehydrogenase [ubiquinone] 1 α subcomplex assembly factor 2 (NDUFA12), or proteins involved in glycolysis, such as fructose-bisphosphate aldolase A (ALDOA) and glucose-6-phosphate isomerase (GPI), were upregulated in the duodenum (Figure 4D). On the other hand, NADH dehydrogenase [ubiquinone] 1 α subcomplex subunit 11 (NDUFA11), NADH dehydrogenase [ubiquinone] iron–sulfur protein 6 (NDUFS6), ATP-citrate synthase (ACLY), and hexokinase-2 (HK2) were downregulated in the jejunum and ileum (Figure 4E,F), suggesting differentially regulated metabolism in different parts of the SI of Lal KO mice.

Next, we examined the activation or inhibition of the identified metabolic pathways using IPA analysis, which included only proteins with significant differences (*p* < 0.05). This analysis revealed a metabolic shift in the duodenum of Lal KO mice (Figure 4G and Table S5). As already indicated by the volcano plots, the TCA cycle (*z*-score = 2.65), oxidative phosphorylation (*z*-score = 1.94), and glycolysis (*z*-score = 1.51) were significantly upregulated in Lal KO compared to the WT duodenum. This switch was not evident or even reversed in the jejunum and ileum because oxidative phosphorylation (jejunum *z*-score = −5.20; ileum *z*-score = −3.00) and TCA cycle (jejunum *z*-score = −3.16) were significantly downregulated in these segments (Figure 4H,I and Tables S6 and S7). To confirm the activation or inhibition of these pathways, we analyzed the abundance of some proteins involved in oxidative phosphorylation in all three intestinal tracts. Consistent with IPA predictions, the respective proteins were upregulated in the duodenum (Figure 4J) but downregulated in the other two parts of the Lal KO mice (Figure 4K,L). These data indicate that only the Lal KO duodenum, the site most affected by lipid and macrophage accumulation, is subject to metabolic remodeling.

### Loss of LAL Triggers a Trem2-like Phenotype in Infiltrating Macrophages throughout the SI

**Figure 5:**
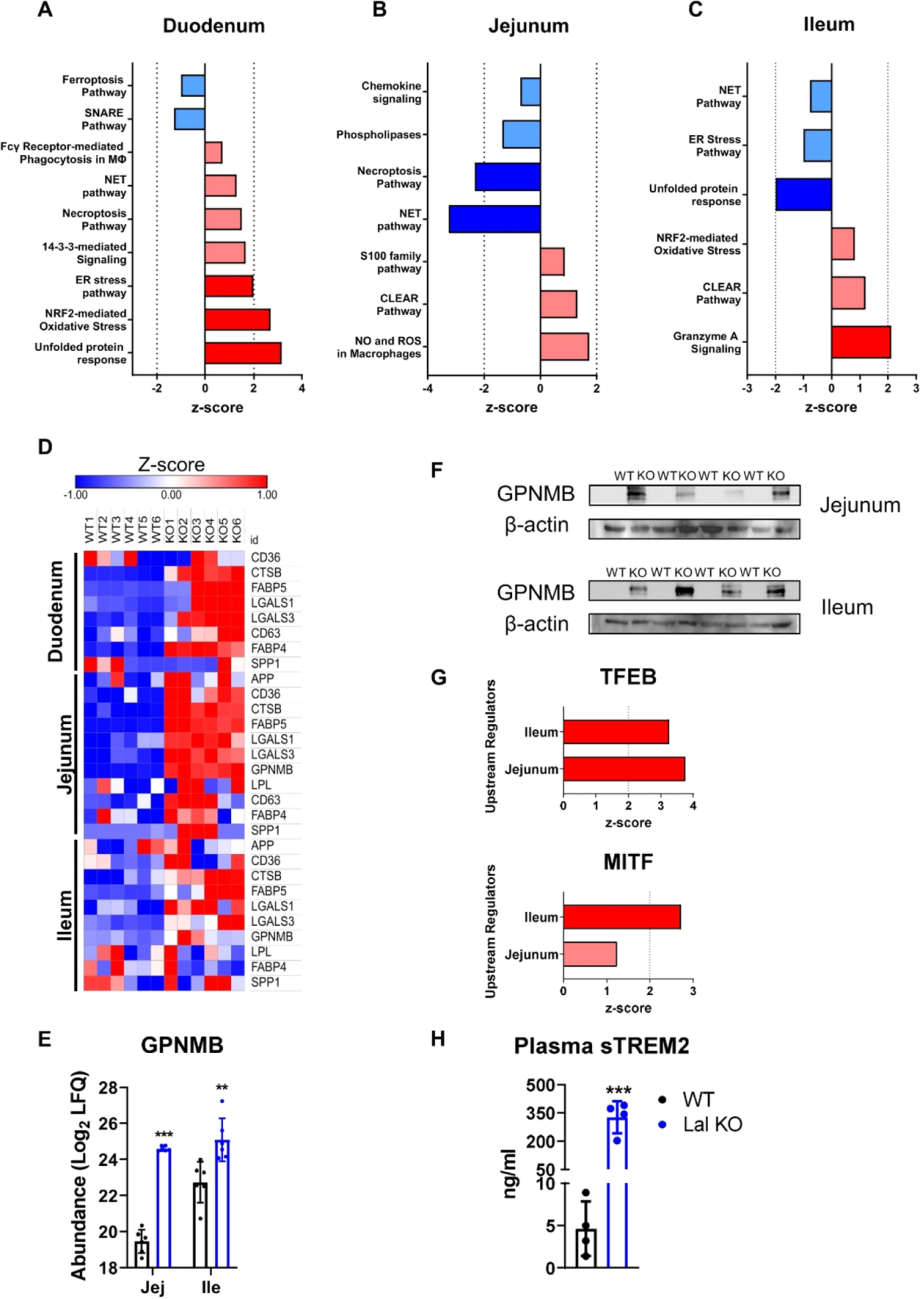
Loss of LAL triggers a Trem2-like phenotype in infiltrating macrophages in the SI of Lal KO mice. Ingenuity pathway analysis (IPA) of enriched canonical pathways related to inflammation of differentially expressed proteins in the (A) duodenum, (B) jejunum, and (C) ileum of Lal KO mice. Dark blue represents pathways with a *z*-score < −2; light blue represents pathways with −2 < *z*-score <0; light red represents pathways with 0 < *z*-score <2; dark red represents pathways with a *z*-score >2. (D) Heatmap of proteins included in the Trem2 signature in the duodenum, jejunum, and ileum of Lal KO mice. (E) Log_2_ values of the LFQ abundances and (F) western blotting analysis of GPNMB protein expression in the jejunum and ileum of WT and Lal KO mice. (G) Activation score of the upstream regulators TFEB and MITF predicted by IPA analysis. Light red represents a *z*-score <2; dark red represents a *z*-score >2. (H) Quantification of circulating sTREM2 in WT and Lal KO mice by ELISA. Data represent mean ± SD (*n* = 4–6); **p ≤ 0.01; ***p ≤ 0.001.

Since we observed increased macrophage infiltration in the SI of Lal KO mice and as these cells play a critical role in immunity and inflammation, we further studied the pathways associated with inflammation and stress response. In particular, the duodenum of Lal KO mice showed activation of the ER stress pathway (*z*-score = 2.00), unfolded protein response (UPR) (*z*-score = 3.16), and macrophage-related pathways, such as necroptosis (*z*-score = 1.51) and phagocytosis (*z*-score = 0.71; Figure 5A). In the jejunum of Lal KO mice, oxidative species production (*z*-score = 1.732) and the lysosomal CLEAR pathway (*z*-score = 1.3) were upregulated (Figure 5B). We also found a comparable upregulation of the CLEAR pathway (*z*-score = 1.213) in Lal KO ilea (Figure 5C), suggesting that the entire SI of Lal KO mice was inflamed.

Using IPA analysis, we identified triggering receptor expressed on myeloid cells-2 (TREM2) as an upstream regulator previously described as an anti-inflammatory marker for a specific subset of macrophages.^15,16,28^ In accordance with the upregulation of *Trem2* in the duodenum of Lal KO mice,^14^*Trem2* expression was also significantly upregulated in the jejunum and ileum of Lal KO mice (Figure S7). This finding was consistent with the prediction analysis of upstream regulators by IPA, suggesting that TREM2 may be implicated in attempts to reduce SI inflammation in Lal KO mice. We therefore examined the abundance of the various proteins involved in the TREM2 signature, such as amyloid-β precursor protein, cluster of differentiation 36 (CD36), CTSB, FABP5, galectin-1 (LGALS1), LGALS3, CD63 antigen (CD63), FABP4, osteopontin (SPP1), and lipoprotein lipase (LPL). The majority of these proteins were consistently upregulated in all three intestinal tracts of Lal KO mice (Figure 5D). The ileum of Lal KO mice showed a less pronounced Trem2-like phenotype, probably because it had the lowest macrophage accumulation. Of note, the TREM2 signature protein glycoprotein nonmetastatic gene B (GPNMB), a previously described marker for various LSDs,^29−31^ was strongly upregulated in the jejunum and ileum of LAL KO mice (Figure 5E,F). In addition, microphthalmia-associated transcription factor (MITF) and transcription factor EB (TFEB), reported as regulators of *Gpnmb* expression,^32^ were predicted to be significantly activated in these parts of the Lal KO SI (Figure 5G), confirming the upregulation of the protein itself.

TREM2 can be proteolytically processed by ADAM proteases and its ectodomain released as a soluble form (sTREM2) into the extracellular milieu.^33^ We therefore measured circulating sTREM2 concentrations, which were increased 71-fold in Lal KO mice (Figure 5H), consistent with *Trem2* mRNA expression (Figure S7) and prediction analysis of proteins conferring a Trem2-like signature to macrophages (Figure 5D). These results indicate that TREM2 is cleaved in Lal KO tissues and released into the circulation.

In summary, these results demonstrate that infiltrating macrophages in the inflammatory environment of the SI of Lal KO mice adopt a Trem2-like phenotype. Moreover, we identified sTREM2 as a possible circulating marker for the systemic inflammation observed in Lal KO mice and possibly GPNMB as a new biomarker for LAL-D.

## Discussion

To the best of our knowledge, we analyzed for the first time the differences and similarities between the duodenum, jejunum, and ileum of WT mice and identified common and segment-specific proteins. We present comprehensive insights into the regional proteome characteristics of the SI of WT mice. Of the 1930 common proteins, only 19 were equally expressed in all three intestinal segments, which are involved in mitochondrial functions, metabolic pathways, as well as complement and coagulation cascades to regulate the metabolic switch during cellular differentiation and to meet the overall defense mechanism against pathogens in the SI. The remaining 1911 proteins and the >500 proteins specific to each segment may explain the different metabolic functions of duodenum, jejunum, and ileum. In addition to our previous correlation of lipase activity profiles throughout the SI,^5^ these results provide a basis for exploring the unique metabolic roles played by the different intestinal tracts in maintaining physiological functionality along the mouse SI. As previous human data have suggested a new and unanticipated role of the ileum in intestinal metabolism,^3^ we reanalyzed the published scRNA-seq data as a pseudobulk to include all cell types and compared the results to our proteomics analysis in the mouse intestinal sections. Most proteins particularly abundant in the mouse ileum correlated with high gene expression in the human ileum. Since human data are usually highly variable due to differences in feeding status, microbiota, and dietary habits, we found a strong correlation between the mouse and human intestinal expression profiles despite the small sample size of the human study. Therefore, we propose that the ileum may play a novel and previously unexpected role in many metabolic pathways.

In addition, we examined in detail the consequences of LAL loss in mice on the proteome signature throughout the SI. The mechanism underlying the intestinal phenotype in LAL-D is poorly understood and could be of great benefit to patients suffering from this disease. In accordance with human data,^3^ we observed a gradual increase in LAL expression along the SI of WT mice. Loss of the enzyme resulted in profound morphological effects on the SI compared to WT controls and also between the three intestinal parts of Lal KO mice. Consistent with our recent publication,^14^ the intestinal segments of Lal KO mice accumulated lipids in different amounts, with the majority of TG in duodenal macrophages, whereas the jejunum was rich in CE crystals. These structural changes resulted in markedly different proteomes between the Lal KO and WT mice. The upregulation of several lysosome-associated proteins, such as cathepsins, throughout the SI of Lal KO mice may be due to a compensatory mechanism by which the cells counteract lipid accumulation. Indeed, increases in cathepsins (CTSD, CTSB, CTSS, CTSK) were reported in various mouse models and in patients suffering from LSDs^34,35^ that were attributed to lipid-laden macrophages characteristic of these diseases. We have recently demonstrated that enterocyte-specific deletion of LAL cannot replicate the intestinal phenotype of global Lal KO mice, suggesting that LAL has a minor effect on enterocyte metabolism.^14^ Thus, although we did not isolate individual cells, we hypothesize that all of the changes in the proteome of Lal KO mice originate from infiltrating macrophages. As the duodenum was metabolically more diverse and active compared to the other two intestinal tracts of Lal KO mice in terms of glycolysis and the TCA cycle, this would imply an upregulation of several metabolic pathways exclusively in duodenal macrophages. Foamy macrophages trigger ER stress and the UPR due to the accumulation of lipids and lipoproteins.^36,37^ Only the duodenum of Lal KO mice with the highest amount of lipids and immune cells activates the UPR, suggesting that infiltrating macrophages at this site may share characteristics similar to those of atherosclerotic foam cells. In contrast, in the ileum of Lal KO mice, ER stress and UPR were even downregulated, possibly reflecting the less pronounced lipid accumulation.

We identified TREM2 as a common upstream regulator throughout the SI of the Lal KO mice. TREM2 is a single-pass transmembrane receptor of the immunoglobulin superfamily that is essential for maintaining the metabolic fitness of macrophages during stress events. Variations in TREM2 were reported to increase the risk of developing late-onset Alzheimer’s disease.^28^ In general, TREM2 plays a protective role by preventing neuroinflammation through downregulating MAPK and NK-κB signaling pathways in the brain,^38^ reducing adipocyte hypertrophy, systemic hypercholesterolemia, inflammation, and glucose intolerance in obesity,^15^ and regulating cholesterol accumulation in foam cells.^39^ Of note, the LAL-encoding *Lipa* gene was included in the gene signature that identifies Trem2^+^ macrophages.^15,40^ In adipose tissue, this signature and, in particular, *Trem2* expression, was detected only in macrophages but in no other immune cell type.^15^ The fact that several proteins belonging to the Trem2 signature cluster were strongly upregulated in the SI of Lal KO mice may suggest that macrophages infiltrate the entire SI and adopt the Trem2 signature to counteract massive lipid accumulation. However, in the absence of LAL, this function is impaired, and sTREM2 is released into the circulation. sTREM2 is elevated in the plasma and cerebrospinal fluid of patients with neurologic inflammatory diseases,^41,42^ nonalcoholic fatty liver disease,^43^ and coronary atherosclerosis,^44^ reflecting the infiltration of monocytes and macrophages in these diseases. Therefore, the extreme increase in the level of sTREM2 in Lal KO mice (and possibly in LAL-D patients) may be a novel marker for the systemic inflammation present in LAL-D.

Among the upregulated proteins, particularly in the jejunum and ileum, we also identified GPNMB, a type 1 transmembrane glycoprotein that has been associated with endosomal/lysosomal compartments in phagocytes.^45,46^ Elevated *Gpnmb* expression was found to be associated with increased numbers of foamy macrophages in human and mouse diseases.^32,47,48^ In agreement with our findings, *Gpnmb* is strongly induced by MITF during lysosomal stress in adipose tissue macrophages of obese individuals and mice.^32^ Although GPNMB is associated with many LSDs and concomitant lysosomal dysfunction, an increase in the level of GPNMB expression in LAL-D has never been reported. The increased expression of the main transcription factors of *Gpnmb* in the SI and also in the liver^49^ of LAL KO mice strongly suggests that this may be a robust marker for identifying LAL-D patients.

Despite the overall inflammation in the SI of Lal KO mice, only duodenal macrophages of Lal KO mice showed upregulation of various mitochondrial-related processes, such as the TCA cycle, oxidative phosphorylation, and glycolysis, to utilize other energy sources because lipids entrapped in lysosomes are not available as a substrate. Deletion of the main microglial pH-regulating protein, Na/H exchanger 1 (NHE1), results in similar upregulation of genes in Trem2-like microglia.^50^ In contrast, jejunal and ileal macrophages exhibited a downregulation of these processes, especially oxidative phosphorylation. These results suggest that only Trem2^+^ macrophages, which are massively overloaded with lipids, activate the aforementioned metabolic rearrangement. The underlying reasons for the distinct metabolic profiles of jejunal and ileal macrophages require further investigation.

## Conclusions

We conclude that regional differences and similarities in the SI proteome of WT mice are comparable to those in gene expression in humans. Despite morphological and functional abnormalities throughout the SI of Lal KO mice, an overall inflamed SI, and the conserved Trem2-like signature, only the duodenum was metabolically more active, as reflected by an increased TCA cycle, glycolysis, and oxidative phosphorylation. This might be a consequence of the enormous amount of lipids entrapped in the lysosomes. Our study raises the possibility that GPNMB and sTREM2 may be used as markers for LAL-D and/or systemic inflammation in LAL-D, which should be investigated in LAL-D patients in the future.

## Data Availability

The mass spectrometry proteomics data sets are available at the ProteomeXchange Consortium via the PRIDE partner repository^51^ under the data set identifier PXD048378. The scRNA-seq data were obtained from the Gene Expression Omnibus database (accession code GSE185224).

## Supporting Information Available

Figure S1. Comparison of proteomes of the duodenum, jejunum, and ileum of WT mice. Figure S2. Abundance of oxidative phosphorylation and lysosomal proteins in the duodenum, jejunum, and ileum of WT mice. Figure S3. Differences and similarities in WT SI upon comparison of two tracts each. Figure S4. Top 20 most abundant proteins in the duodenum, jejunum, and ileum of WT mice. Figure S5. Comparable intestinal expression profile in mice and humans. Figure S6. Proteome analysis of the three intestinal tracts from WT and Lal KO mice. Figure S7. Increased *Trem2* mRNA expression in the jejunum and ileum of WT and Lal KO mice. (PDF)
Table S1. Proteome and KEGG enrichment of intestinal segments of WT mice. (XLSX)
Table S2. Common proteins and KEGG enrichment of comparison of two tracts of WT mice. (XLSX)
Table S3. Specific proteins to each intestinal segment of WT mice. (XLSX)
Table S4. scRNA-seq counts of the three intestinal segments of healthy humans. (XLSX)
Table S5. Proteome, KEGG enrichment, and IPA prediction of the duodenum of WT and Lal KO mice. (XLSX)
Table S6. Proteome, KEGG enrichment, and IPA prediction of the jejunum of WT and Lal KO mice. (XLSX)
Table S7. Proteome, KEGG enrichment, and IPA prediction of the ileum of WT and Lal KO mice. (XLSX)

## Abbreviations

CE: cholesteryl ester
FDR: false discovery rate
GO: Gene Ontology
GPNMB: glycoprotein nonmetastatic gene B
KEGG: Kyoto Encyclopedia of Genes and Genomes
LAL: lysosomal acid lipase
LAL-D: lysosomal acid lipase deficiency
Lal KO: lysosomal acid lipase-deficient
LAM: lipid-associated macrophage
LFQ: label-free quantification
Lipa: LAL-encoding gene
LSD: lysosomal storage disorder
scRNA-seq: single-cell RNA sequencing
SI: small intestine
Trem2: triggering receptor expressed on myeloid cells-2
TG: triacylglycerol
UPR: unfolded protein response
