# Supplementary material for: Regional Differences in the Small Intestinal Proteome of Control Mice and of Mice Lacking Lysosomal Acid Lipase
Source: J Proteome Res. Author manuscript; Available in PMC 2024 Apr 5. (PMC7615810; doi:10.1021/acs.jproteome.4c00082)
Supplement: Fig. S1-S7 [file EMS194559-supplement-Fig__S1_S7.pdf]

Supporting information

Regional differences in the small intestinal proteome of control mice and of mice lacking lysosomal acid lipase

Valentina Bianco[1], Monika Svecla[2], Giovanni Battista Vingiani[2], Dagmar Kolb[3,4,5], Birgit Schwarz[1], Martin Buerger[1], Giangiacomo Beretta[6], Giuseppe Danilo Norata[2,7,*], and Dagmar Kratky[1,4,*]

[1]Gottfried Schatz Research Center, Molecular Biology and Biochemistry, Medical University of Graz, Graz, Austria

[2]Department of Pharmacological and Biomolecular Sciences, Università degli Studi di Milano, Milan, Italy

[3]Core Facility Ultrastructural Analysis, Medical University of Graz, Graz, Austria

[4]BioTechMed-Graz, Graz, Austria

[5]Gottfried Schatz Research Center, Cell Biology, Histology and Embryology, Medical University of Graz, Graz, Austria

[6]Department of Environmental Science and Policy, Università degli Studi di Milano, Milan, Italy

[7]Centro SISA per lo studio dell'Aterosclerosi, Ospedale Bassini, Cinisello Balsamo, Italy

*correspondence: Dagmar Kratky, Gottfried Schatz Research Center, Molecular Biology and Biochemistry, Medical University of Graz, Neue Stiftingtalstrasse 6/4, 8010 Graz, Austria; phone: +43 316 385 71965, e-mail: dagmar.kratky@medunigraz.at; Giuseppe Danilo Norata, Department of Pharmacological and Biomolecular Sciences, Università degli Studi di Milano, Via Balzaretti 9, 20133, Milan, Italy; phone: +390250318402; e-mail: danilo.norata@unimi.it

Table of Content

Supplemental Figures

Figure S1. Comparison of proteomes of duodenum, jejunum, and ileum of WT mice.

Figure S2. Abundance of oxidative phosphorylation and lysosomal proteins in duodenum, jejunum, and ileum of WT mice.

Figure S3. Differences and similarities in WT SI upon comparison of two tracts each.

Figure S4. Top 20 most abundant proteins in the duodenum, jejunum, and ileum of WT mice.

Figure S5. Comparable intestinal expression profiles in mice and humans

Figure S6. Proteome analysis of the three intestinal tracts from WT and Lal KO mice.

Figure S7. Increased and GPNMB protein and *Trem2* mRNA expression in the jejunum and ileum of WT and Lal KO mice.

Supplemental Tables

Table S1. Proteome and KEGG enrichment of intestinal segments of WT mice.

Table S2. Common proteins and KEGG enrichment in two-tract comparison of WT mice.

Table S3. Specific proteins to each intestinal segment of WT mice.

Table S4. scRNA-seq counts of the three intestinal segments of healthy humans.

Table S5. Proteome, KEGG enrichment, and IPA prediction of duodenum of WT and Lal KO mice.

Table S6. Proteome, KEGG enrichment, and IPA prediction of jejunum of WT and Lal KO mice.

Table S7. Proteome, KEGG enrichment, and IPA prediction of ileum of WT and Lal KO mice.

Supplemental Figures

Figure S1

A

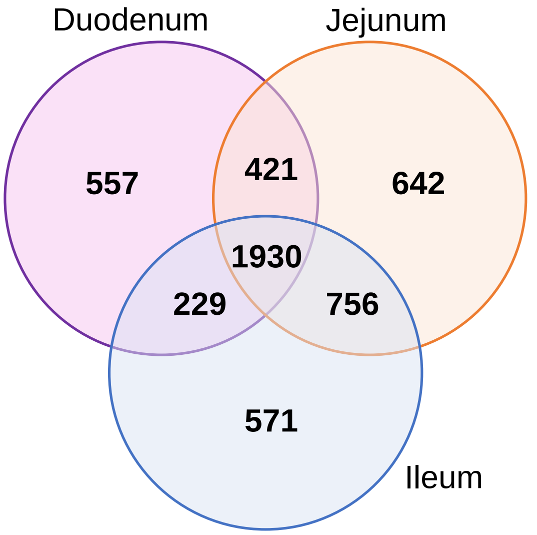

B

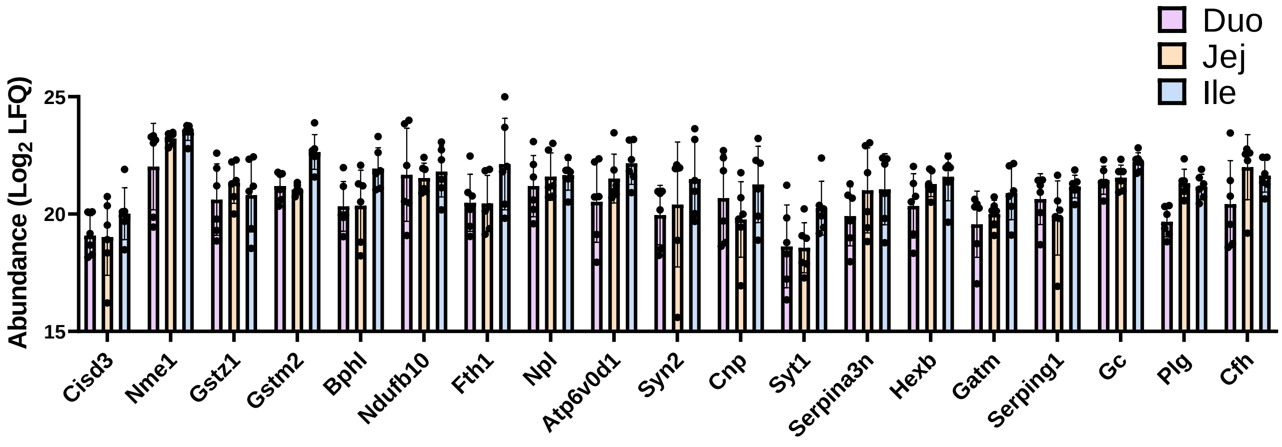

Figure S1. Comparison of proteomes of duodenum, jejunum, and ileum of WT mice. (**A**) Venn diagram of the proteome of duodenum, jejunum, and ileum of WT mice. (**B**) Log2 abundance of nonsignificantly different proteins in the SI of WT mice. Data represent means ± SD (n = 6).

Figure S2

A

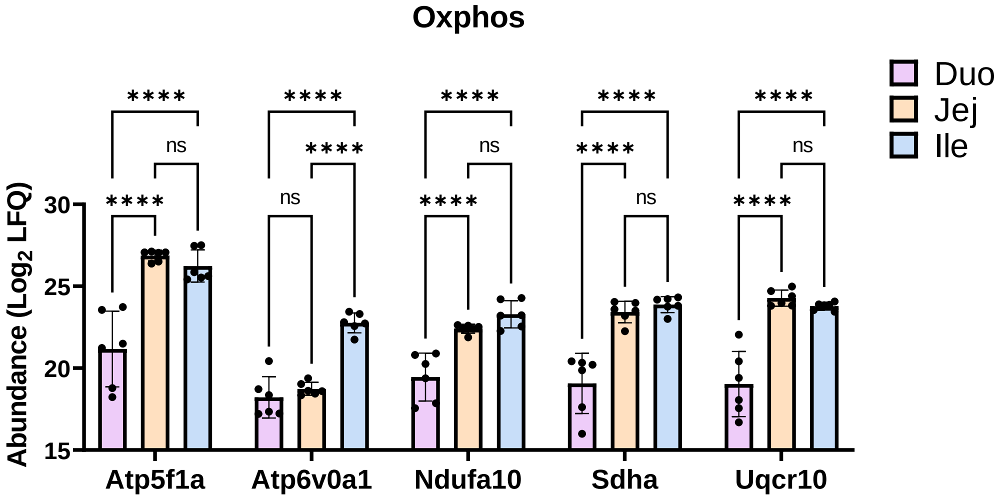

B

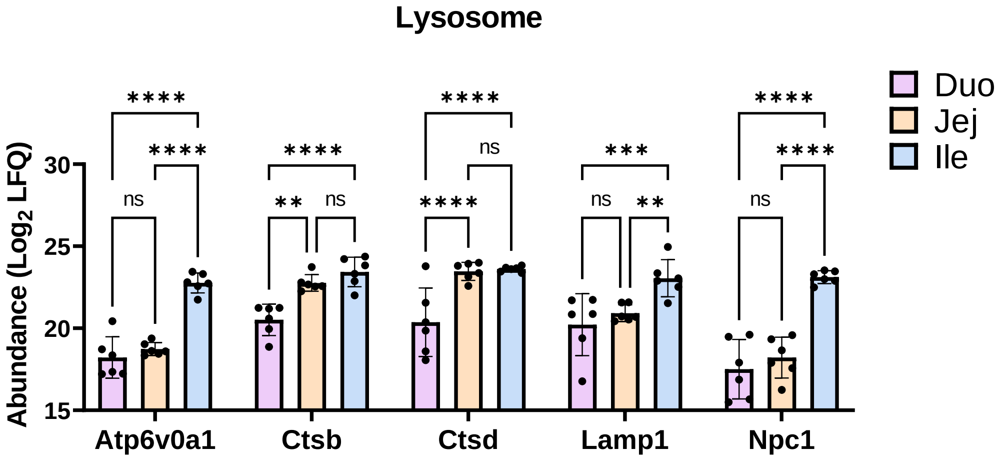

Figure S2. Abundance of oxidative phosphorylation and lysosomal proteins in duodenum, jejunum, and ileum of WT mice. Log_2 values of the LFQ abundances of several proteins involved in (**A**) oxidative phosphorylation and (**B**) lysosome. Data represent means ± SD. (n = 6); ** $p \leq 0.01$; *** $p \leq 0.001$, **** $p \leq 0.0001$

Figure S3

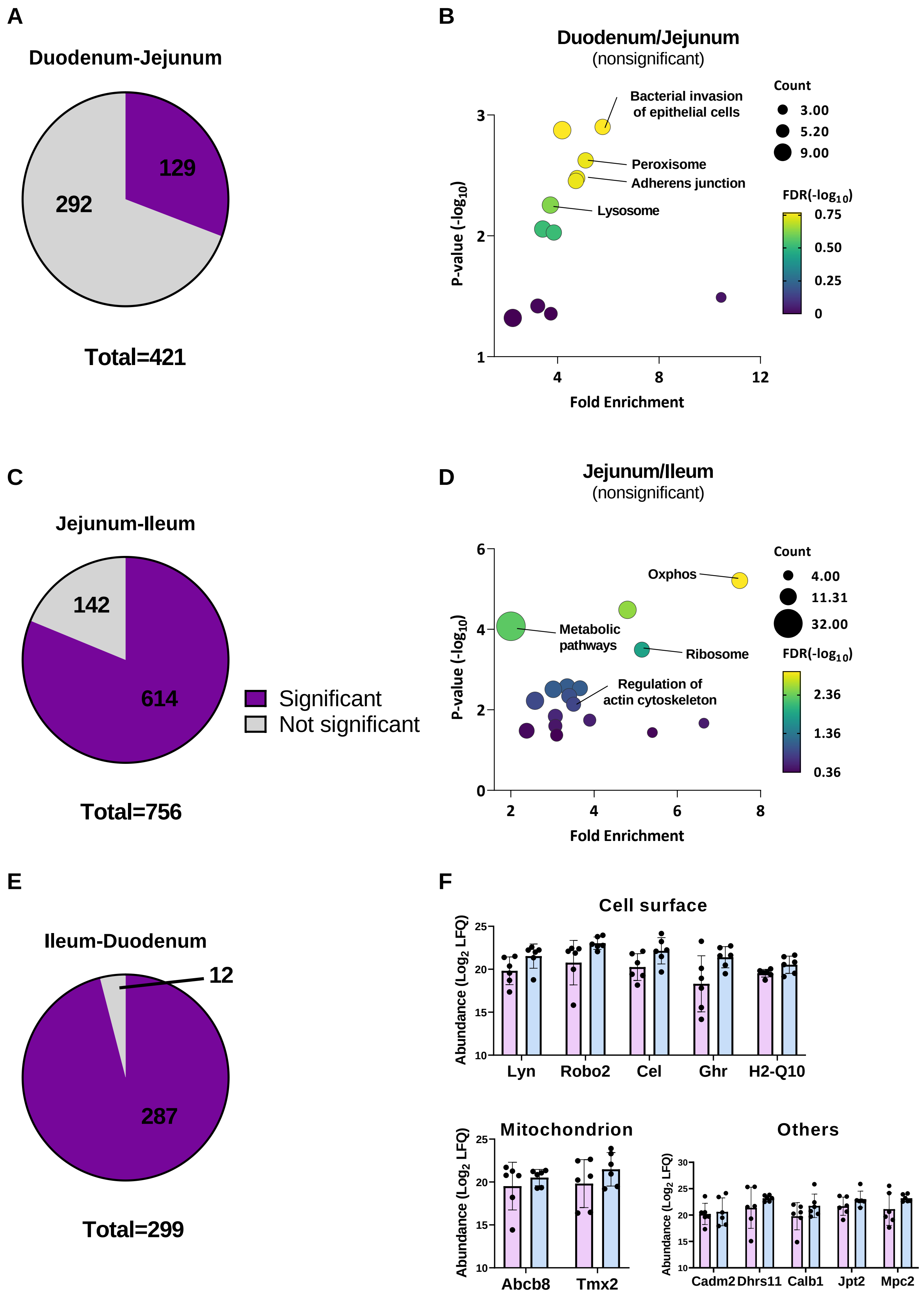
A
Duodenum-Jejunum
129
292
Total=421
B
Duodenum/Jejunum
(nonsignificant)
Bacterial invasion of epithelial cells
Peroxisome
Adherens junction
Lysosome
Count
3.00
5.20
9.00
FDR(-log10)
0.75
0.50
0.25
0
P-value (-log10)
Fold Enrichment
C
Jejunum-Ileum
142
614
Total=756
Significant
Not significant
D
Jejunum/Ileum
(nonsignificant)
Oxphos
Metabolic pathways
Ribosome
Regulation of actin cytoskeleton
Count
4.00
11.31
32.00
FDR(-log10)
2.36
1.36
0.36
P-value (-log10)
Fold Enrichment
E
Ileum-Duodenum
12
287
Total=299
F
Cell surface
Abundance (Log2 LFQ)
Lyn
Robo2
Cel
Ghr
H2-Q10
Mitochondrion
Abcb8
Tmx2
Others
Cadm2
Dhrs11
Calb1
Jpt2
Mpc2

Figure S3. Differences and similarities in WT SI upon comparison of two tracts each. Pie chart representing the significant and non-significant proteins in two-tract comparison between (**A**) duodenum and jejunum, (**C**) jejunum and ileum, and (**E**) ileum and duodenum. Bubble plots of KEGG analysis of non-significantly differentially expressed proteins between (**B**) duodenum and jejunum, (**D**) jejunum and ileum, and (**F**) duodenum and ileum of WT mice.

Figure S4

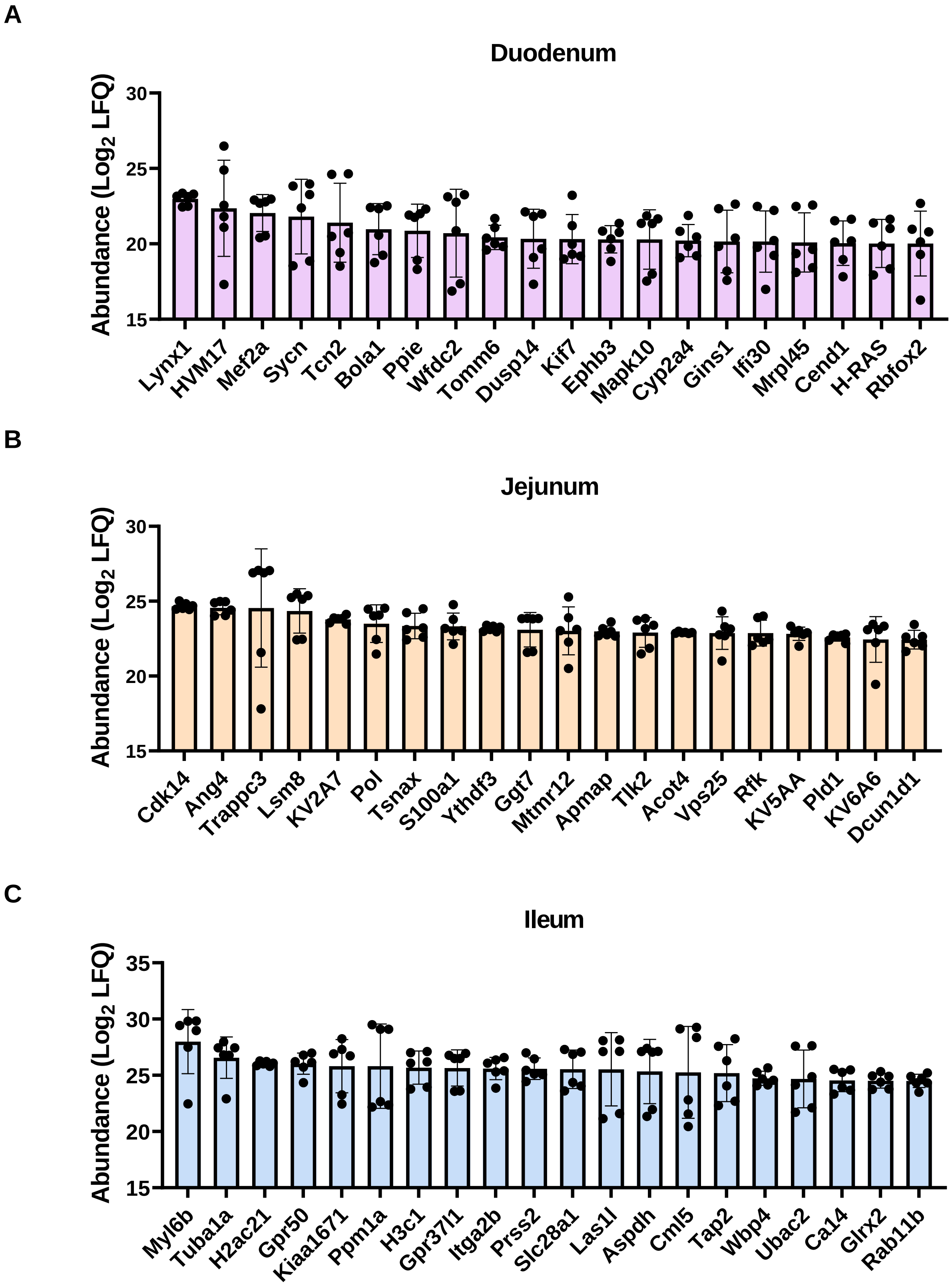

Figure S4. Top 20 most abundant proteins in the duodenum, jejunum, and ileum of WT mice. Log_2 values of the LFQ abundances of the 20 most abundant proteins in the (**A**) duodenum, (**B**) jejunum, and (**C**) ileum of WT mice. Data represent means ± SD (n = 6).

Figure S5

A

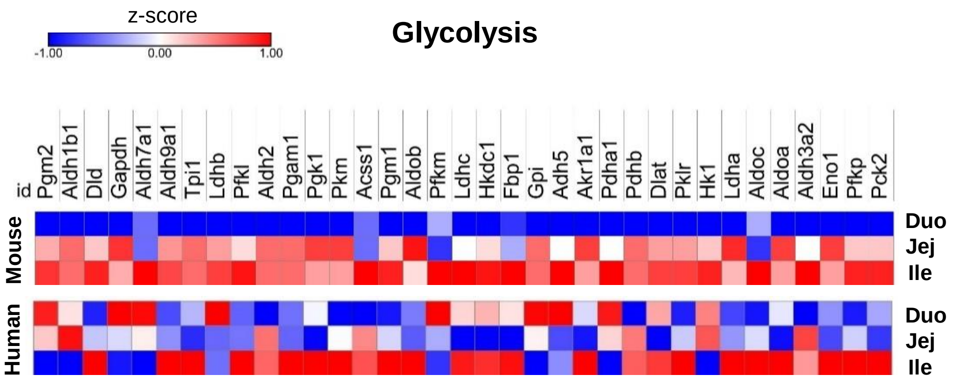

B

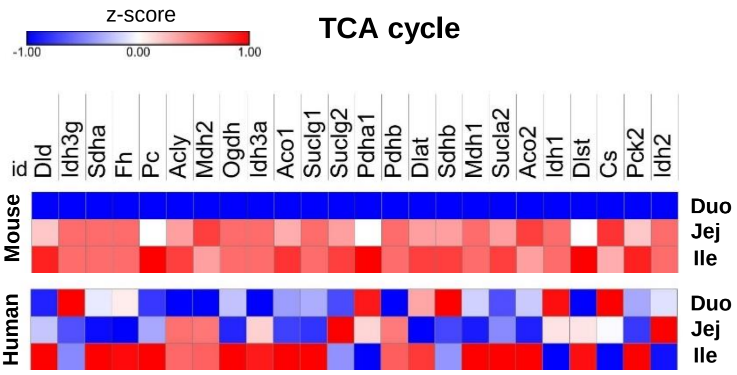

C

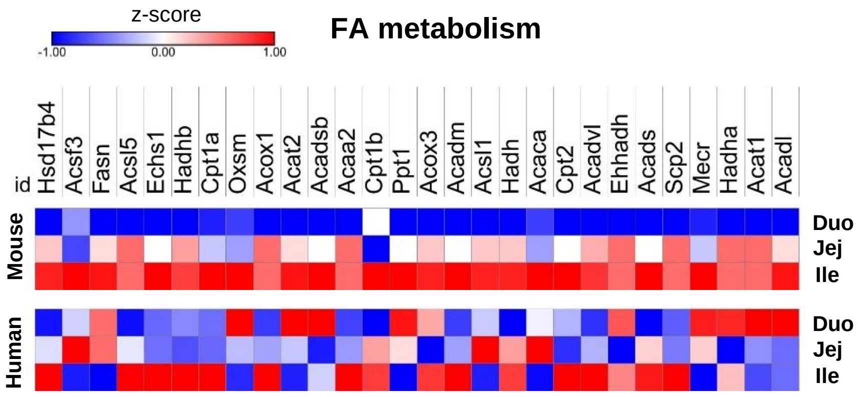

Figure S5. Comparable intestinal expression profile in mice and humans. Heatmaps of proteins (mouse, top) and genes (human, bottom) involved in pathways related to (**A**) glycolysis, (**B**) TCA cycle, and (**C**) FA metabolism.

Figure S6

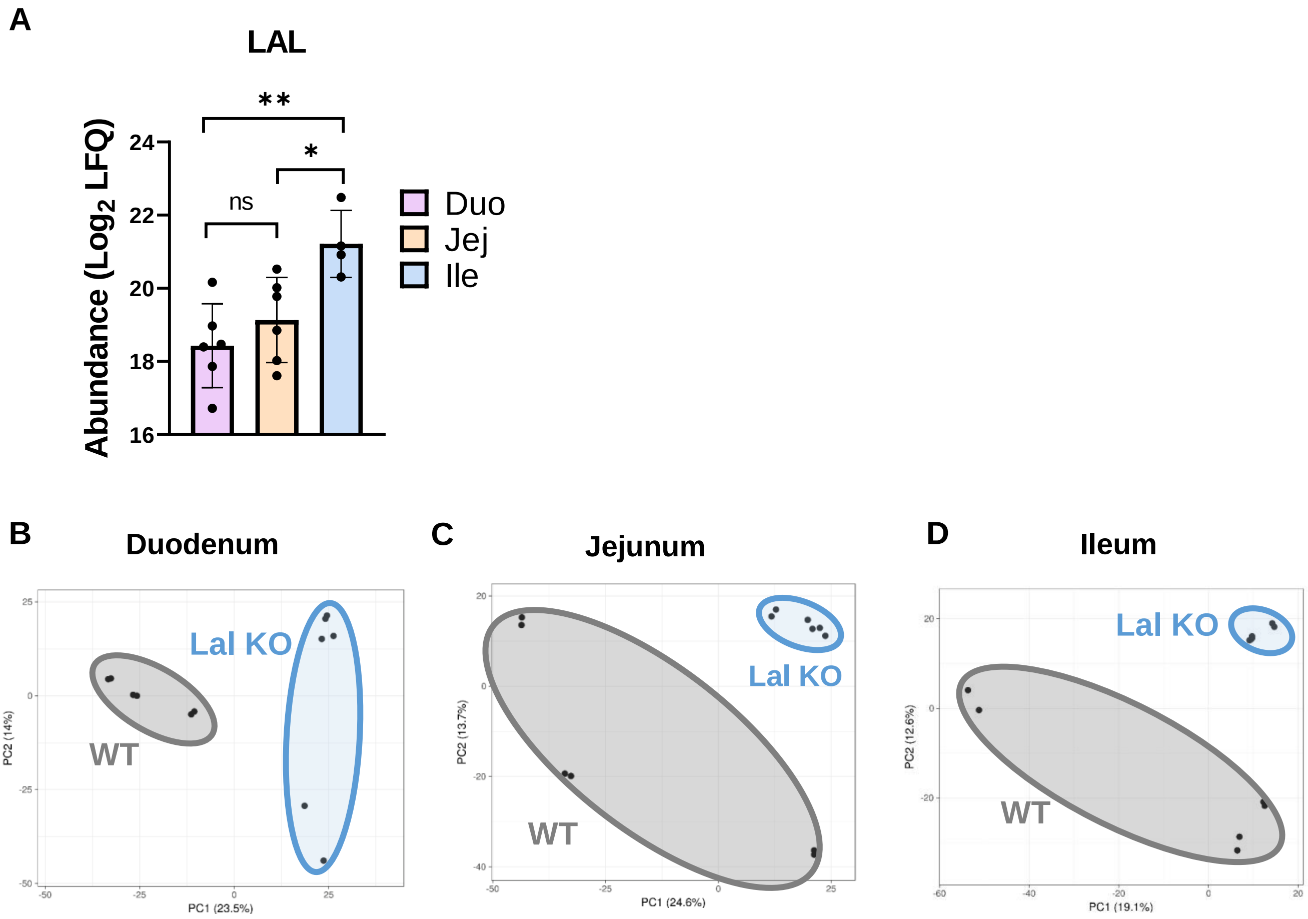

Figure S6. Proteome analysis of the three intestinal tracts from WT and Lal KO mice. (**A**) Log_2 values of the LFQ abundances of LAL in the SI of WT mice. PCA plots of the proteome of (**B**) duodenum, (**C**) jejunum, and (**D**) ileum of WT and Lal KO mice. Data represent means ± SD. (n = 4-6); * $p < 0.05$, ** $p \leq 0.01$.

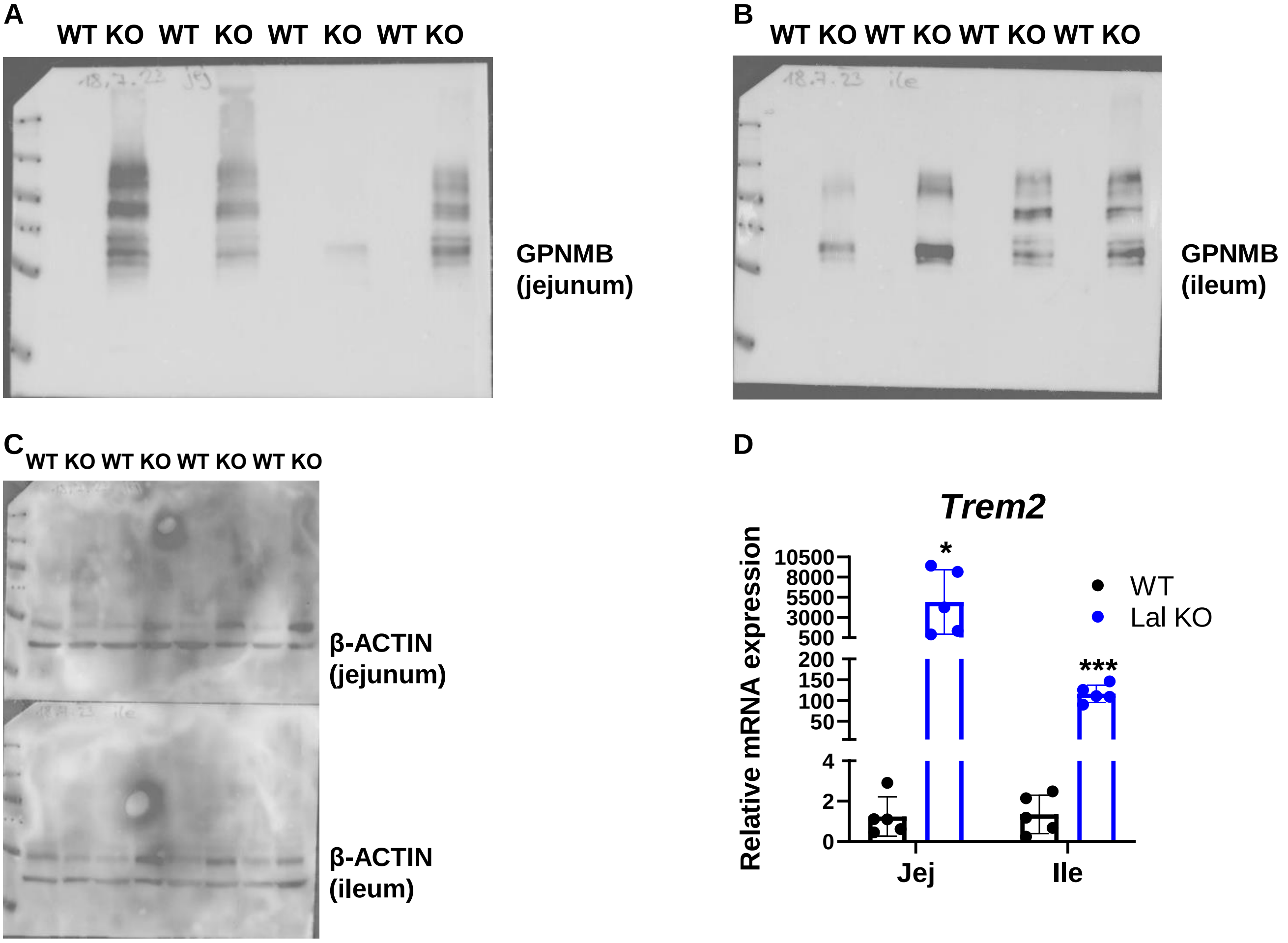

Figure S7. Increased GPNMB protein and *Trem2* mRNA expression in the jejunum and ileum of WT and Lal KO mice. Western blotting analysis of GPNMB protein expression in (**A**) jejunum and (**B**) ileum of WT and Lal KO mice. (**C**) Protein expression of β-actin was used as loading control. (**D**) *Trem2* mRNA expression in the jejunum and ileum. Data represent means ± SD (n = 5); * $p < 0.05$, *** $p \leq 0.001$.
